# Muscle Mass Measurement Using Machine Learning Algorithms with Electrical Impedance Myography

**DOI:** 10.3390/s22083087

**Published:** 2022-04-18

**Authors:** Kuo-Sheng Cheng, Ya-Ling Su, Li-Chieh Kuo, Tai-Hua Yang, Chia-Lin Lee, Wenxi Chen, Shing-Hong Liu

**Affiliations:** 1 Department of Biomedical Engineering, National Cheng Kung University, Tainai 701, Taiwan; kscheng@mail.ncku.edu.tw (K.-S.C.); ya1993225@gmail.com (Y.-L.S.); dd2006tw@gmail.com (T.-H.Y.); 2 Department of Occupational Therapy, National Cheng Kung University, Tainan 701, Taiwan; jkkuo@mail.ncku.edu.tw; 3 Department of Physical Education, National Kaohsiung Normal University, Kaohsiung City 80201, Taiwan; lee.ort@gmail.com; 4 Biomedical Information Engineering Laboratory, The University of Aizu, Aizu-Wakamatsu City, Fukushima 965-8580, Japan; wenxi@u-aizu.ac.jp; 5 Department of Computer Science and Information Engineering, Chaoyang University of Technology, Taichung 413310, Taiwan

**Keywords:** sarcopenia, electronic impedance myography, mass of thigh muscle, ridge regression, support vector regression

## Abstract

Sarcopenia is a wild chronic disease among elderly people. Although it does not entail a life-threatening risk, it will increase the adverse risk due to the associated unsteady gait, fall, fractures, and functional disability. The import factors in diagnosing sarcopenia are muscle mass and strength. The examination of muscle mass must be carried in the clinic. However, the loss of muscle mass can be improved by rehabilitation that can be performed in non-medical environments. Electronic impedance myography (EIM) can measure some parameters of muscles that have the correlations with muscle mass and strength. The goal of this study is to use machine learning algorithms to estimate the total mass of thigh muscles (MoTM) with the parameters of EIM and body information. We explored the seven major muscles of lower limbs. The feature selection methods, including recursive feature elimination (RFE) and feature combination, were used to select the optimal features based on the ridge regression (RR) and support vector regression (SVR) models. The optimal features were the resistance of rectus femoris normalized by the thigh circumference, phase of tibialis anterior combined with the gender, and body information, height, and weight. There were 96 subjects involved in this study. The performances of estimating the MoTM used the regression coefficient (*r^2^*) and root-mean-square error (RMSE), which were 0.800 and 0.929, and 1.432 kg and 0.980 kg for RR and SVR models, respectively. Thus, the proposed method could have the potential to support people examining their muscle mass in non-medical environments.

## 1. Introduction

The lifespan of the world’s population is increasing and society is gradually aging. According to the report of the United Nations, the number of elderly people (over 65 years of age) in the world in 2019 was 703 million, and this is estimated to double to 1.5 billion by 2050 [1]. In Taiwan, the report of the National Development Council indicated that the elderly population over 65 years of age will exceed 20% of the national population in 2026 [2]. For healthy adults, aging results in a progressive loss of muscle mass and strength. According to the study of Kim and Choi, people over forty years would have 8% loss of muscle mass every decennium. When their ages are over seventy years, the muscle mass loss would be 15% every decennium [3]. Although the loss of muscle mass and strength is interdependent, the loss of muscle strength occurs at least 2–4 times faster than the loss of muscle mass. Its behavior is also a more important risk factor for predicting adverse outcomes, such as unsteady gait, fall, fractures, and even the increased risk of disability and death [4]. Many studies have shown that the decline of lower limb muscles is greater than that of the upper limb [5,6,7]. Thus, sarcopenia is currently a hot topic for the aging society, which is defined as the progressive reduction in muscle mass in elderly people [8], and serves as the physical functional criteria, such as low muscle strength and low physical performance [9,10].

There are some diagnostic criteria for sarcopenia provided by the European Working Group on Sarcopenia in Older People, International Working Group on Sarcopenia and Asian Working Group for Sarcopenia. The examined items include the muscle mass, muscle strength, and physical performance, which are carried out step by step. The different working groups advocate the different criteria [11]. There are three main techniques used to estimate skeletal muscle mass, including computed tomography (CT) [12], magnetic resonance imaging (MRI) [13], and dual energy X-ray absorptiometry (DXA) [14,15]. However, these techniques all require professional operators to perform the examination in clinical practice. Therefore, how to develop a measurement method used in non-medical place will be a challenging issue.

Electromyography (EMG) measures the electrical activity in motor neurons and muscle fibers during muscle contraction, which allows for the early diagnosis of neuromuscular diseases, including neuropathies, myopathies, and motor neuron diseases [16,17]. Because the morphology of the EMG signal during the isotonic or isometric contractions represents the size and shape of muscle fiber territories, the characteristics of the time or frequency domain of EMG signal are used to evaluate the muscle condition [18,19,20]. The effect of aging on the EMG amplitude during dynamic contractions presents a sufficient difference [21]. Tian et al. studied the performances of EMG and mechanomyography in detecting age-related sarcopenia [22]. Leone et al. used the morphology of EMG signal to evaluate the levels of sarcopenia [23]. However, the EMG measurement requires the performance of muscle contraction via electrical stimulation or voluntary activity. Thus, patients will feel uncomfortable. 

Bioelectrical impedance analysis has been used in many physiological measurements, and it is a physical measure of the ionic conduction of a specific body segment in contrast with electrical conduction characteristics [24,25]. Bioimpedance is a complex quantity composed of the resistance caused by total body water and reactance caused by the capacitance of the cells [26]. The change of the reactance in the blood is modulated by the orientation of red cells being changed when the blood flows [27]. Thus, it could be used to measure the hemodynamic parameters, e.g., blood flow, stroke volume, and thoracic fluid. Impedance cardiography is the most important application [28]. Liu et al. used impedance plethysmography (IPG) to perform cuffless blood pressure measurement [29]. Some previous studies used this technique to measure the mass of the interested muscle, which is called the electrical impedance myography (EIM) [30]. Tanaka et al. used EIM to measure the skeletal muscle mass in the limb segment in comparison to measure via the MRI method. There was a standard error of 6.1% [31]. Rutkove et al. used EIM to evaluate amyotrophic lateral sclerosis (ALS), which had a high relation with the hand-held dynamometry (correlation coefficient (*r*) of 0.82) and the ALS Functional Rating Scale Revised (*r* of 0.74) [32]. Kortman et al. found that the dependency among phase of EIM, age, and gender for the skeletal muscle was high [5]. 

Machine learning (ML) algorithms are considered promising approaches for clinical prediction and classification problems, which include convolutional neural networks, support vector machines, random forests, and many others [33,34]. An ML method primarily includes the feature engineering, appropriate ML algorithm, training and evaluation of model performance, and using the trained model to predict the unknown data [35,36]. The feature processing is an important issue that can directly affect the performance of an ML algorithm. The more accurate the features, the higher the performance of the ML algorithm. Liu et al used a multilayer neural network to estimate cuffless blood pressure [37]. Mahajan et al. used logistic regression and random forest to evaluate heart failure [38]. Kwon et al. applied the one-dimension convolution neural network to estimate the change of stroke volume with the blood pressure waveform [39]. Although ML methods have been popularly used for clinical prediction for some issues, some traditional statistical analysis methods have also reignited interest in exploiting these fields [40,41].

This study aims to estimate the total mass of thigh muscles (MoTM) by EIM with ML algorithms. Seven muscles of lower limbs were measured, including rectus femoris, vastus lateralis, medial femoris, tibialis anterior, semitendinosus, biceps femoris, and gastrocnemius. The parameters of EIM were the impedance, resistance, reactance, and phase, and the body information included age, weight, body mass index (BMI), gender, thigh circumference, and calf circumference. Thus, the number of total parameters was thirty-seven. Recursive feature elimination (RFE) was used to select the important parameters as the features for ML input. Two ML models, namely ridge regression (RR) and support vector regression (SVR), were used, and their performances were verified by the data from ninety-six subjects.

## 2. Materials and Methods

**Figure 1:**
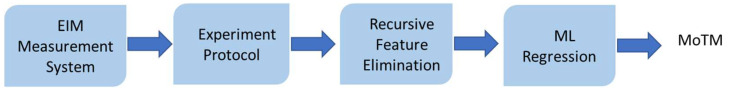
The framework of this study. A measurement system is used to measure the parameters of EIM for the muscles of lower limb. According to the experiment protocol, we recruited ninety-six subjects. RFE is used to select the important features to estimate the total MoTM by the ML models.

Figure 1 shows the framework of this study. An EIM measurement system was developed, which includes an impedance measurement module and a data acquisition board. A graphic user interface (GUI) was also designed to display and record the EIM signals. According to the guide for the examination of sarcopenia [11], we recruited 96 subjects to evaluate the skeletal muscular mass of their lower limbs. The optimal parameters were determined by recursive feature elimination. Finally, two ML models used these parameters to estimate the total MoTM.

### 2.1. EIM Measurement System

An impedance measurement module (BIOPAC EP 100, BIOPAC® System, Goleta, CA, USA) was used to measure four parameters of the interesting skeletal muscles. A DAQ board (NI DAQ USB-6361, National Instruments, Austin, TX, USA) was used to acquire the EIM signals, i.e., phase and impedance.

#### 2.1.1. Calibration of EIM Measurement System

**Figure 2:**
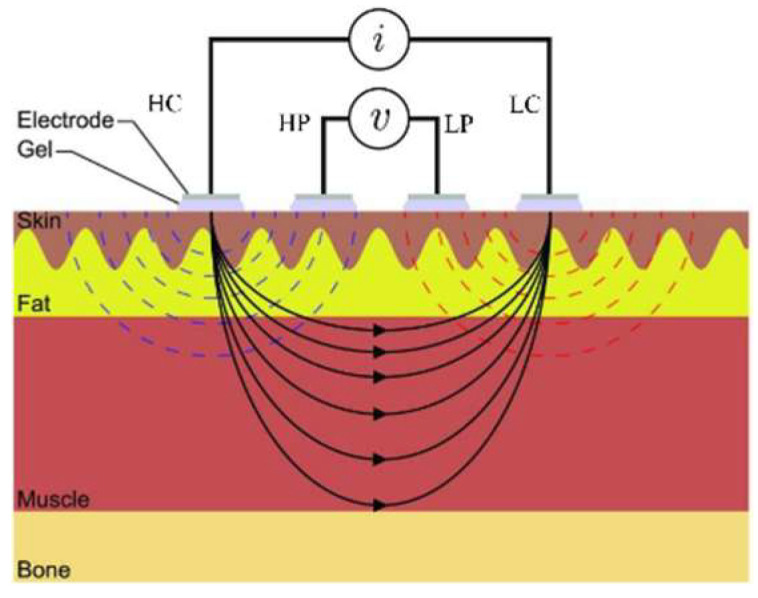
Placements of four electrodes and the distribution of electric field under the EIM measurement.

The sampling rate was 500 Hz in the EIM measurement system. The input alternating current was 50 kHz in frequency and 0.4 mA (root mean square, RMS). The impedance sensitivity was 100 Ω/voltage, and the phase sensitivity was 9°/voltage. The cutoff frequency of low-pass filter was 10 Hz. Figure 2 shows the placement of four electrodes and the distribution of the electric field. The four electrodes include two current electrodes (positive and negative terminals, HC and LC) and two voltage electrodes (positive and negative terminals, HP and LP). The parameters of EIM are resistance (*R*), reactance (Z), phase (*P*), and impedance (*I*). The relation among *R*, *Z*, P, and *I* is defined below,

(1) $$I=\sqrt{R^{2}+Z^{2}}$$

(2) $$P=tan^{-1}\frac{Z}{R}$$

**Figure 3:**
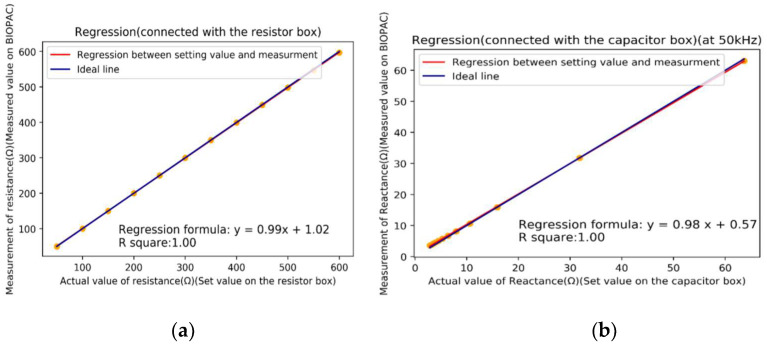
Calibrations of BIOPAC EP 100 module. (**a**) Calibration of resistance with a resister box. (**b**) Calibration of reactance with a capacitor box.

The output signals of the EP 100 module are impedance *I* and phase *P*. According to Equations (1) and (2), Z and *R* can be calculated by the measured *I* and *P*. We calibrated the impedance measurement module with a resistor box and a capacitor box, respectively. Figure 3a shows the calibration of resistance. The dots indicate the measured points, and the red line is the practical calibrated line (regression line) approximated by the measured points. The blue line is the designed ideal line. The mean square error was 0.052 Ω, and the square of the correlation coefficient *r^2^* was 1.00. Equation (3) shows the calibrating function of the resistor:

(3) y=0.99x+1.02,

where *x* is actual resistance (Ω) and y is the measured resistance (Ω). Figure 3b shows the calibration of reactance. The red line is the calibrated line, and the blue line is the designed ideal line. The mean square error was 0.042 Ω, and *r^2^* was 1.00. Equation (4) shows the calibrating function of the resistor,

(4) y=0.98x+0.57,

where *x* is actual reactance (Ω), and y is the measured reactance (Ω).

#### 2.1.2. Placement of Electrodes

**Figure 4:**
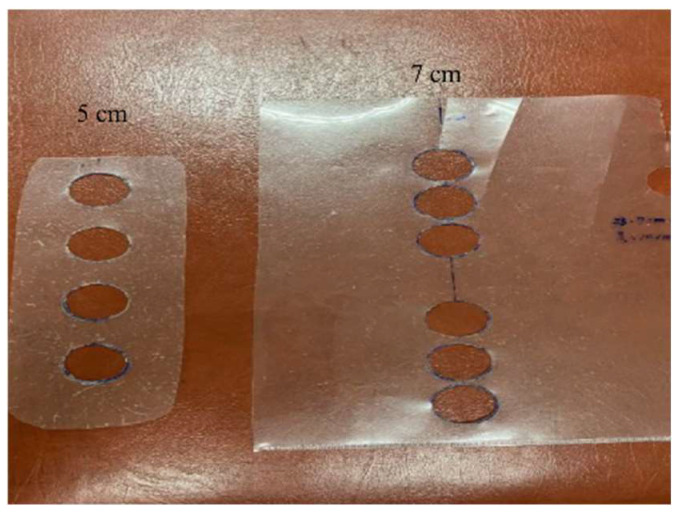
Placement of four electrodes with two schemes, 5 cm and 7 cm.

Sanchez et al. suggested that the error of placement of electrodes in the EIM would affect the reproducibility according to the intraclass correlation coefficient (ICC) [30]. The larger the ICC, the smaller the error rate of the distance between the two electrodes. Moreover, the four electrodes must be aligned. In this study, we defined the placement of four electrodes with two schemes, 5 cm and 7 cm in length, to fit for the larger and smaller muscles, respectively. We used the translucent tapes to make the markers for electrode positioning, as shown in Figure 4. The length of the left tape is 5 cm, and that of the right one is 7 cm.

### 2.2. Experiment Protocol

The potential subjects underwent the hand-grip strength and walk test before participation in the experiment. The participants had to perform 28 kg and 18 kg grips for the male and female subjects, respectively. Moreover, their walking speeds must be over 0.8 m/second. There were 96 subjects participating in this study, and the number of male and female subjects was 42 and 54, respectively. The information of subjects is shown in Table 1, which includes age, height, weight, BMI, as well as thigh and calf circumferences. The experiment protocol was approved by the Institutional Review Board, National Cheng Kung University Hospital (NCKUH). Written informed consent was obtained from each participant prior to entering the test procedure. (B-ER-108-126)

According to the previous studies, the masses of lower limb muscles declined easier than those of upper limb muscles when people have sarcopenia [5,6,42]. Thus, we measured the tibialis anterior and gastrocnemius in the calf muscles, and vastus lateralis, rectus femoris, medial famous, biceps femoris, and semitendinosus in the thigh muscles. Table 2 shows the landmarks of each muscle. The vastus lateralis, medial famous, and tibialis anterior muscles belong to the small muscles, the other muscles belong to the large muscles.

A subject was requested to comfortably lie supine on a table whose face was upward. We measured the thigh and calf circumferences. Then, the total MoTM was measured by the InBody S10 (InBody Co. Ltd. Korea) as the reference. Next, the subject was asked to maintain the same posture. Four BIOPAC EP 100 modules were used synchronously to measure the masses of vastus lateralis, rectus femoris, medial femoris, and tibialis anterior at approximately 60 s. Finally, the subject was requested to change their posture with face downward. Three BIOPAC EP 100 modules were used synchronously to measure the masses of biceps femoris, semitendinosus, and gastrocnemius.

### 2.3. Extracting Features

**Figure 5:**
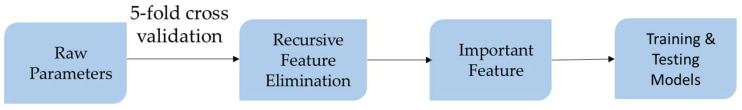
Flowchart of extracting features.

Figure 5 shows the flowchart of extracting features. The RFE is used to search the optimal parameters [43,44] as the features to estimate the MoTM. The parameters of 85 subjects randomly selected from the 96 subjects are the raw data. In order to reduce the flag problems, such as overfitting or selection bias, the RFE uses the five-fold cross validation to evaluate the optimal parameters. Table 3 shows the used parameters of subjects that not only include the EIM parameters, but also contain the body information of subjects. Thus, there are 34 parameters. RFE fitted a model to remove the weakest features until the specified number of features was reached. All features were ranked by *r^2^* of the model, and by recursively eliminating a feature with the lowest coefficient per loop. The lower the impact feature, the lower the change of coefficient. Thus, RFE could eliminate the features with the dependencies and collinearity existing in the model. 

### 2.4. Machine Learning Models

The traditional regression problem usually uses the linear multiple-regression method that fits the regression curve as close to the training data as possible. This would cause the testing data to contain an amount of error, which is called the overfitting problem, if the input variables are highly correlated. Therefore, the training regression curve should not be too close to the training data, so that the predictions have better results. In this study, we used two machine learning models with the concept of error margin, RR, and SVR, to estimate MoTM. 

#### 2.4.1. Ridge Regression

A linear multiple-regression model is written below,

(5) $$\hat{y}=\overset{p}{\underset{j=1}{\sum }}\beta_{j}x_{j}+\varepsilon$$

where $\hat{y}$ is the estimated value, *x_j_* is the independent variable, *β_j_* is the coefficient, *p* is the number of independent variables, and ε is residual error. A loss function (L) will be defined by the regression function, as in Equation (6),

(6) $$L\left(y,f\left(x\right)\right)=\frac{1}{N}\overset{N}{\underset{i=1}{\sum }}L\left(y_{i},f\left(x_{i}\right)\right)$$

The sum square error (SSE) is usually used as a loss function, and the object is to minimize the loss function to estimate the *β_j_*,

(7) $$minimize\{SEE=\overset{N}{\underset{i}{\sum }}{\left(y_{i}-\hat{y_{i}}\right)}^{2}\}$$

where *y_i_* is the observed value and *N* is the number of observed values. Ridge regression (RR) adds the penalty parameter to the objective function [45],

(8) $$minimize\{SEE+\lambda \overset{p}{\underset{j}{\sum }}\beta_{j}{}^{2}\}$$

Because this parameter is a second-order penalty for the coefficient, it is also called the L2 penalty parameter. The value of the L2 penalty parameter can be controlled by λ. When λ approaches 0, Equation (8) is equal with Equation (7). When λ approaches to the infinite, all coefficients approach 0. In this study, λ is set to 0.1.

#### 2.4.2. Support Vector Regression

The difference between SVR and linear regression is that an error margin is acceptable to find an appropriate model to fit the data. In Equation (9), the error term (ε) is instead handled in the constraints, where the absolute error is less than or equal to a specified margin, called the maximum error,

(9) $$\left|y_{i}-\hat{y_{i}}\right|\leq \varepsilon$$

Thus, a loss function (*L*) is defined by a regression function (*f*) and adds the constraint, a specified margin. However, this margin could not comprise all of the data. Some of the data still fall outside the margin. Therefore, a slack variable is defined such that any data falling outside of ϵ is denoted its deviation from the margin as ξ. The objective function can add the slack variable below,

(10) $$minimize\{L(y,\;f\left(x\right)+C\overset{N}{\underset{i=1}{\sum }}\left|\xi_{i}\right|\}$$

The margin is changed as,

(11) $$\left|y_{i}-\hat{y_{i}}\right|\leq \varepsilon +\left|\xi_{i}\right|$$

Moreover, SVR can use the different kernel functions, linear or nonlinear functions, to convert the nonlinear data distribution to the linear distribution [46]. In this study, the kernel function is a 2nd-order polynomial function, *C* is set to 0.1, and *ξ* to 0.3.

## 3. Results

The results of this study included the optimal features and MoTM estimation. For the search of optimal features, we not only studied the impact of each parameter, but also combined the complementary parameters to yield a more significant feature set. Then, the performances of MoTM estimation by RR and SVR models were compared.

### 3.1. Optimal Feature Sets

According to the description in Section 2.3, the impact of each feature depends on the regression models. After the RFE process, we only chose the parameters with positive weight coefficients. Table 4 shows the ranks and weight coefficients of these parameters under RR and SVR. There are nine and eight parameters for RR and SVR, respectively. We used theses parameters as the feature sets to train RR and SVR models, whose regression coefficient (*r^2^*) were 0.812 and 0.831, separately.

The gender parameter is the categorical variable, and TC and CC are the geometric variables which are affected by the bone, tissue, fat, and muscle. Thus, we combined these parameters with EIM parameters to yield a more substantial feature set. From Table 4, the major thigh muscles are the rectus femoris and vastus laterals, and the major calf muscles are the gastrocnemius and tibialis anterior. The new EIM features for the thigh muscles were the RF_R, RF_Z, and VL_Z parameters normalized by the TC parameter, which individually added to the original feature vectors to train the RR and SVR models. Table 5 shows the regression coefficient (*r^2^*) for the RR and SVR models. The RF_R/TC has the best performance for RR and SVR models, whose *r^2^* values increase to 0.816 and 0.840.

The new EIM features for the calf muscles were the TA_P and GT_P parameters combining with the gender parameter, i.e., the means of TA_P and GT_P for the male and female groups separately multiplying with the TA_P and GT_P in the male and female groups. These new features were individually added to the original feature vectors to train the RR and SVR models. Table 6 shows the regression coefficient (*r^2^*) for the RR and SVR models. The TA_P_Gender has the best performance for RR and SVR models, whose *r^2^* values increase to 0.825 and 0.840.

### 3.2. Performance of Regression Models

In Table 4, the body information are the height, weight, and gender. However, the gender parameter has been combined with the EIM parameter. Thus, we chose the height and weight as the features. Moreover, in Table 5 and Table 6, the RF_R/TC and TA_P_Gender features have the best performance. Thus, we also chose these two features. Then, the final features were height, weight, RF_R/TC, and TA_P_Gender, which were used to train the RR and SVR models again. The subjects who were not used to train the models were used as the testing data. The number of testing samples was 11. The regression coefficients (*r^2^*) of RR and SVR models were 0.800 and 0.929, and the RMSEs were 1.432 kg and 0.980 kg, respectively.

## 4. Discussion

According to the study of Chen et al., the sarcopenia diagnosis not only measures the skeletal muscle mass and strength, but also tests some physical performances [11]. The measurement of skeletal muscle mass implies significant costs in terms of clinical practice. Although some commercial apparatus with bioelectrical impedance analysis (BIA) technology can measure the global muscle mass, its price and size are hard to accept in the context of homecare. The benefits of EIM are that it can measure the electrical parameters of single muscle and its operation is much easier than BIA. Thus, when the injured muscle is improved with rehabilitation, EIM could measure the real change of this muscle [47]. In this study, an apparatus, InBody S10, with the BIA technology was used to measure the total MoTM. However, we only measured the RF_R and TA_P parameters with the EIM method and body information to estimate the MoTM based on the RR and SVR models. The regression coefficients (*r^2^*) between two methods were 0.800 and 0.929, respectively.

According to the study of Janssen et al., the parameters of estimating skeletal muscle mass were the height, age, gender, and resistance of BIA [48]. The regression coefficients (*r^2^*) approached 0.86. In our study, we proposed 34 parameters, including the body information of subject (excluding age) and parameters of EIM, as shown in Table 3. Different machine learning algorithms may perform properly with different feature sets, even if they are using the same training set [49]. Therefore, we used the RFE method to rank these parameters for RR and SVR models. In Table 4, the height, weight, gender, and RF_R are the common parameters for two models. This result was very close to the previous study. 

For the traditional regression estimation, the categorical parameters, such as gender, country, or race, are difficult to utilize because they are only encoded. Therefore, the regression functions depend on the different categorical parameters to increase the estimating accuracy [5,50]. In Table 4, the impact ranking of gender parameter is only second based on RR and SVR models. Thus, the performance of RR and SVR models built on the different genders could not be improved. The external direct product in the group theorem is a general method to process the data of two groups [51]. Thus, we used this method to reinforce the differences of EIM parameters for the different genders. 

Theoretically, more features should result in better discriminating performance, but the practical experience for the machine learning algorithms shows this doctrine not to be applicable for many cases [37,52,53,54]. A regression model with more features would possibly reduce modeling bias. However, its predicting accuracy would decrease, and its computational complexity would increase. Hence, we used RFE to select the optimal feature set. The number of features for RR and SVR models was reduced to 10 and nine. Moreover, another method with dimensionality reduction, also known as feature extraction, usually uses the linear combination with the given features to reduce the size of feature space without losing information of the original feature space [55]. In this study, we tried to combine the parameters of EIM with the body information, including thigh circumference and gender. In Table 5 and Table 6, RF_R_TC and TA_P_Gender features significantly increase the performance of RR and SVR models. Thus, we only selected four features, namely height, weight, RF_R_TC, and TA_P_Gender, to estimate the total MoTM. The SVR model had the better performance, with a regression coefficient (*r^2^*) of 0.929 and RMSE of 0.98 kg.

Aaron et al. [56] and Tarulli [57] found the relation between the phase parameter of EIM measuring the TA and GT muscles and age and muscle atrophy. In Table 4, the TA_P and GT_P parameters are the important features for the RR and SVR models. Moreover, Kortman et al. found that the changes of resistance and reactance parameters of EIM for the skeletal muscle would be affected by the age and gender of subjects [5]. In Table 4, RF_R and RF_Z parameters also are the important features.

There are three reasons for the loss of mass, namely a reduction in the number of muscle fibers, shrinking in the size of muscle fibers, and transformation of muscle fibers into type I fibers. The different conditions will make the changes of the different parameters of EIM. For the change of muscle fiber number, the resistance parameter of EIM will increase, and the phase parameter will decrease. For the change of muscle fiber size, the intracellular fluid of muscle will decrease, which would cause a drop in the phase parameter of EIM. In this study, subjects, excluding those have injurious lower limbs and muscle weakness, generally have one of the three conditions listed above. The proposed method only used the resistance and phase parameters of EIM to estimate the muscle mass of lower limbs. Thus, this is considered as the limitation of this study.

Moreover, the BIA method includes the EIM and IPG, which not only measures the impedance of muscles, but also measures the tissue components including the fat, vessel wall, skin, etc., and the blood flow. Wróbel et al. proposed a pulse-dynamics analysis to calibrate these potential error parameters [58]. The pulse signal can be measured by the IPG [29]. Thus, in the future, the EIM signal could be calibrated by the dynamic pulse signal measured by IPG to reduce the distortions induced by lipids in the skin of various thickness for individual patients. 

## 5. Conclusions

Sarcopenia is a prevalent disease for elderly people when their limbs or vertebra are injured. Moreover, the muscle mass usually decreases with age. Thus, people could avoid the loss of muscle mass with therapy for physical fitness. The development of a measurement system used in a non-medical environment could support people with the hidden risk of sarcopenia to examine their muscle mass condition every day. In this study, the contribution is to utilize the data mining technique, extracting important features, such as the resistance and phase parameters of EIM, and body information, and using two machine learning algorithms, namely RR and SVR, for estimating the total MoTM. Their regression coefficient (*r^2^*) and RMSE all are better than previous studies. Thus, the proposed method has the potential for screening skeletal muscle mass in non-medical environments in the future.

## Figures and Tables

**Table 1:** The information of subjects.

	Total (*N* = 96)	Male (*N* = 42)	Female (*N* = 54)
Age (years)	48.29 ± 17.91	44.31 ± 18.24	51.39 ± 17.18
Height (cm)	162.68 ± 7.51	168.93 ± 5.13	157.82 ± 5.07
Weight (Kg)	64.75 ± 11.64	71.10 ± 10.66	59.81 ± 9.91
BMI (Kg/m^2^)	24.40 ± 3.64	24.90 ± 3.38	24.01 ± 3.82
Thigh circumference (cm)	50.02 ± 5.34	50.41 ± 5.23	49.71 ± 5.45
Calf circumference (cm)	36.07 ± 3.13	37.07 ± 2.81	35.31 ± 3.16

**Table 2:** The landmarks of seven muscles.

Muscle	Start Point	End Point
Vastus Lateralis	Lateral patella	Greater trochanter
Rectus Femoris	Midline of patella	Anterior superior iliac spine
Medial Femoris	Medial patella	Medial side of femur
Tibialis Anterior (small)	Lateral condyle of tibia	Midline of calf
Semitendinosus (small)	Posterior medial knee joint	Midline of gluteal fold
Biceps Femoris	Posterior lateral knee joint and head of fibula	Midline of gluteal fold
Gastrocnemius	Posterior knee joint	Midline of calf

**Table 3:** Raw parameters including EIM parameters of seven muscles and body information of subjects.

Basic Information	Data Type	EIM Data		Data Type
Height	Numerical	Rectus Femoris (RF)	Impedance (*I*) Phase (P) Resistance (*R*) Reactance (Z)	Numerical
Weight		Vastus Lateralis (VL)		
BMI		Medial Femoris (MF)		
Gender	Categorical	Tibialis Anterior (TA)		
Thigh Circumference (TC)	Numerical	Semitendinosus (ST)		
Calf Circumference (CC)		Biceps Femoris (BF)		
		Gastrocnemius (GT)		

**Table 4:** The ranks and weight coefficients of parameters under the RR and SVR models by the RFE process.

Rank	Ridge Regression		SVR	
	Parameter	Weight Coef. (*r^2^*) Mean ± SD	Parameter	Weight Coef. (*r^2^*) Mean ± SD
1	Height	0.139 ± 0.151	Height	0.194 ± 0.189
2	Gender	0.087 ± 0.134	Gender	0.108 ± 0.166
3	TC	0.040 ± 0.033	RF_R	0.044 ± 0.090
4	RF_R	0.023 ± 0.097	TC	0.028 ± 0.051
5	Weight	0.009 ± 0.031	Weight	0.019 ± 0.030
6	CC	0.009 ± 0.015	GT_P	0.012 ± 0.041
7	RF_Z	0.008 ± 0.019	TA_P	0.009 ± 0.038
8	TA_P	0.001 ± 0.092	CC	0.008 ± 0.026
9	VL_Z	0.000 ± 0.036		

SD: the abbreviation of standard division.

**Table 5:** Regression coefficient (*r^2^*) for the RR and SVR models when adding combined features, RF_Z/TC, RF_R/TC, and VL_Z/TC, separately.

Features	*r^2^* of RR	*r^2^* of SVR
RF_Z/TC	0.817	0.832
RF_R/TC	0.816	0.840
VL_Z/TC	0.815	0.831

**Table 6:** The regression coefficient (*r^2^*) for the RR and SVR models when adding combined features, TA_P_Gender and GT_P_Gender, separately.

Features	*r^2^* of RR	*r^2^* of SVR
TA_P_Gender	0.825	0.840
GT_P_Gender	0.819	0.832

## Abbreviations

EIM	electronic impedance myography
RFE	recursive feature elimination
RR	ridge regression
SVR	support vector regression
RMSE	root-mean-square-error
CT	computed tomography
MRI	magnetic resonance imaging
DXA	dual energy X-ray absorptiometry
EMG	electromyography
IPG	impedance plethysmography
EIM	impedance myography
ALS	amyotrophic lateral sclerosis
ML	machine learning
MoTM	total mass of thigh muscles
BMI	body mass index
REF	recursive feature elimination
GUI	graphic user interface
RMS	root mean square
R	resistance
Z	reactance
P	phase
I	impedance
ICC	intraclass correlation coefficient
TC	thigh circumference
CC	calf circumference
RF	rectus femoris
VL	vastus lateralis
MF	medial femoris
TA	tibialis anterior
ST	semitendinosus
BF	biceps femoris
GT	gastrocnemius
SSE	sum square error
r^2^	regression coefficient
